# Prognostic and Therapeutic Implications of Tumor Biology in Colorectal Liver Metastases

**DOI:** 10.3390/cancers14010088

**Published:** 2021-12-24

**Authors:** Carsten Kamphues, Katharina Beyer, Georgios Antonios Margonis

**Affiliations:** 1 Department of General and Visceral Surgery, Charité Campus Benjamin Franklin, 12203 Berlin, Germany; carsten.kamphues@charite.de (C.K.); katharina.beyer2@charite.de (K.B.); 2 Department of Surgery, Memorial Sloan Kettering Cancer Center, New York, NY 10065, USA

Prognostic models allow clinicians to predict survival outcomes, facilitate patient–physician discussions, and identify subgroups with potentially distinct prognoses. Although such prognostic stratification cannot directly predict treatment benefit, it can help to inform clinical decision making. This editorial will discuss potential avenues for these topics in the context of colorectal cancer liver metastases (CRLM).

Several prognostic variables for CRLM have been described in the literature. The most recent development is the inclusion of *KRAS* mutational status in prognostic models. The use of two other potent biomarkers, *TP53* and *SMAD4*, may further refine prognostication, although tumors are not routinely tested for these somatic mutations [1,2]. In comparison, primary tumor laterality is a surrogate of tumor biology that is readily available but has yet to be included in prognostic scores that include biomarkers. It has the potential to improve prognostication as it has a strong prognostic value that is partially independent from *RAS/RAF* mutations [3]. An alternative approach is to create separate prognostic models for subsets of patients with distinct tumor biologies (e.g., separate models for those with *KRAS*mut vs. wild-type tumors). In fact, the literature has demonstrated that the prognostic value of clinicopathologic factors varies according to different tumor biologies [4,5]. Specifically, prognostic factors gain or lose significance due to the absence or presence of other variables. Relevant examples include the interplay of *KRAS* status with tumor side and margin width [6,7]. Thus, a model trained in “all comers” may perform sub-optimally because it cannot capture the aforementioned heterogeneity. The development of separate prognostic models for patients with distinct tumor biologies can also provide insight into the possible interactions of biomarkers with clinicopathologic factors. A novel methodological approach is to apply machine learning (ML) methods to develop a clinical prediction model. Unfortunately, only a few ML-based prognostic models have been developed for patients with CRLM, and most of them use outdated techniques such as CART. As interpretability is equally important to predictive power, causal forests (an extension of Random Forests), which are causal inference learning methods and modern classification trees, may be more appropriate than “black box” approaches such as gradient boosting [8]. Of note, many of the current prognostic models lack external validation, which can inform whether a model can be generalized outside the cohort that was used to train it. Furthermore, the plethora of new prognostic models that incorporate genetic information mandates studies that will compare model performance [9,10,11,12].

Prognostic models that use biomarkers can also help guide treatment decisions in areas where prospective data are not conclusive, such as optimal patient selection for single stage hepatectomy. For example, although we know that patients with a high GAME or m-CRS score fare relatively poorly, we lack studies that compare the long-term outcomes of these patients to those who received the best medical treatment [10,11]. This question cannot ethically be answered using randomized trials, which is why these scores that harness tumor biology can be useful. Another clinically relevant question is whether optimal margin width can be tailored according to tumor biology. A few studies have reported on the outcomes of patients with R0 vs. R1 resections after stratifying by *KRAS* mutational status, but more studies are needed before we can reach a consensus [13,14]. Furthermore, only a few of these studies have attempted to find the optimal margin cut-off for patients with *KRAS*mut vs. wild-type tumors. Of note, no meta-analysis to date has collectively analyzed these studies, although several have analyzed the outcomes of patients with different margin widths without considering tumor biology [15]. Another grey area of current CRLM management includes whether patients with upfront resectable CRLM should get neoadjuvant chemotherapy and whether patients with resected CRLM should receive adjuvant chemotherapy. The use of prognostic models that include biomarkers may aid in decision making as randomized trials are inconclusive. For example, the value of GAME score in guiding the decision of whether to offer perioperative chemotherapy has been tested in the past; future studies can evaluate the use of other prognostic scores that include biomarkers (e.g., e-CRS and m-CRS) for this purpose [16]. This may be particularly important in light of a recent prospective study from Japan on the lack of benefit from adjuvant chemotherapy in patients with “low risk” disease [17]. Optimizing follow-up strategies for patients with distinct biologies is another unanswered question. Lastly, although repeat hepatectomy is an established approach for patients with a recurrent disease that is amenable to surgery, there is a paucity of prognostic scores that include biomarkers for these patients. This is surprising as previous studies have suggested that the association of *KRAS* with poor outcomes is pronounced in this setting [18]. Thus, the development of prognostic models exclusively in patients with recurrent CRLM could help guide patient selection for second surgeries.

There is also a scarcity of studies assessing the outcomes of patients with *KRAS*mut tumors who undergo two-stage hepatectomies (TSH) as well as the potential role of *KRAS* and other biomarkers in predicting the risk of TSH dropout. Existing models assume that variables interact in a linear and additive fashion and are constructed by assigning points to each variable based on the odds ratio calculated in a logistic regression analysis. Those points are added, and the sum corresponds to a certain risk of dropout. The mathematical realities, however, suggest that the interactions among these factors may be far from linear, and that variables gain or lose significance due to the absence or presence of other variables. In turn, these linear predictive models do not capture these interactions between predictors. The prognostic role of primary tumor laterality in conventional TSH is also largely unknown. However, it has recently been suggested that *KRAS*mut metastases that originate from a right-sided primary portend extremely poor prognosis in patients who undergo a novel form of TSH (ALPPS) [19]. Of note, *KRAS* status has not been shown to be prognostic in patients who undergo liver transplantation (LT). In turn, it may be worth investigating whether the “enhanced” *RAS* mutation (the triple *RAS, SMAD4,* and *TP53* co-mutation) or the G12V *KRAS* point mutation that is associated with particularly poor outcomes can be used to identify patients who may not benefit from LT [20]. Finally, the nature of LT makes it ideal for tumors that recur within the liver but less ideal for tumors that recur outside the liver. Thus, future studies may investigate whether codon specific *KRAS* mutations, which are reportedly associated with an increased risk of extrahepatic recurrences after CRLM resection, predict extrahepatic recurrences following LT [21].

Collectively, this Special Issue welcomes studies that explore the use of biomarkers to refine prognosis and aid in the management of patients who undergo single stage hepatectomy, two stage hepatectomies, and LT for CRLM.
